# Supplementary material for: Clinical Decision-Making for Heart Failure in Kosovo: A Conjoint Analysis
Source: Int J Environ Res Public Health. 2022 Nov 8;19(22):14638. doi: 10.3390/ijerph192214638 (PMC9690698; doi:10.3390/ijerph192214638)
Supplement: Supplementary file 1 [file ijerph-19-14638-s001.zip › ijerph-1899221-supplementary.pdf]

**Table S1. Example case from the first experiment.**
> 70 years old
Smoker
No family history with cardiovascular problems
No comorbidities
BMI 25 - 30 (over-weight)
No dyspnoea
Reduced tolerance to physical activity
Persistent coughing or wheezing
No signs of appetite loss or nausea
Confused
No oedema
Increased heart rate
Orthopnoea
Unusual fatigue
Visible jugular veins in the neck area
□Yes □No

**Table S2. Example case from second experiment.**
Normal jugular venous pressure
Hepato-jugular reflux
No pleural effusion
Displaced point of maximal impulse
Third heart sound can be heard
No heart murmur
Lung insufficiency
CPR 3 - 10 mg/L
Troponin > 0.4 ng/mL (high)
Creatinine kinase - MB 5 - 25 UI/L
LDH 140 - 280 UI/L
BNP < 100 pg/mL
NTproBNP > 400 pg/mL
□Yes □No
